# 3D Total Body Photography as a Promising Innovation for Early Skin Cancer Detection: Scoping Review

**DOI:** 10.2196/68510

**Published:** 2025-12-17

**Authors:** Fran Baete, Alyssa Laura Jakers, Emilie Delanoye, Nele Vande Velde, Griet Voet

**Affiliations:** 1 Clinique Dermatologie Gent, Hippoliet lippensplein 24A, Gent, 9000, Belgium, 32 093353783

**Keywords:** 3D total body imaging, 3D total body photography, artificial intelligence, melanoma, nonmelanoma skin cancer, skin cancer

## Abstract

**Background:**

Skin cancer (SC) is a global health concern because of its high and still increasing incidence and associated health care cost. Belgium is no exception as 1 in 5 people are diagnosed with SC before the age of 75 years. The VECTRA WB360, a 3D total body photography system, allows clinicians to objectively compare the totality of the skin on a macroscopic level on further appointments. The integrated lesion visualization software allows automated detection, counts, and assessment of skin lesions. Detailed comparison of individual lesions is possible through the attached digital dermatoscope.

**Objective:**

This study aims to review available literature on the use of the VECTRA in research and clinical settings and to summarize the clinical utility, advantages, and limitations reported for this system.

**Methods:**

An electronic literature search was conducted on PubMed from December 2023 to March 2024 using a combination of the following search terms: 3D imaging, VECTRA WB360, melanoma, nonmelanoma skin cancer, their synonyms, and associated entry terms. Publications that used a device other than the VECTRA WB360 were excluded, as were papers reporting on new technology without further research or without added cases. After a thorough screening of the papers and removal of duplicates, 11 papers remained.

**Results:**

Our literature search yielded 11 relevant papers, which included 2 case studies, 6 prospective studies, and 3 retrospective studies. According to multiple studies, the VECTRA WB360 images were of a high enough quality to allow on-screen diagnosis of some melanoma and nonmelanoma skin cancers by dermatologists. Sensitivity compared to face-to-face examination for melanoma is unknown. The integrated lesion visualization software is capable of detecting and counting naevi and distinguishing melanoma from other skin lesions with high accuracy, with convolutional neural network integration further enhancing its sensitivity and specificity. However, it is important to note that no comparison to the usual standard of care was made. Also, dermatologists achieved greater specificity and thus remained superior to machine and artificial intelligence (AI).

**Conclusions:**

Although the VECTRA 3D TBP holds substantial promise for the early detection and monitoring of SC, its application cannot yet replace the expertise of trained clinicians. Although the lesion visualizer and dermoscopy explainable intelligence (DEXI) score offer potential enhancements, they also pose risks, including a significant increase in unnecessary excisions due to lower specificity. Expert overview is still recommended and superior, since there is not enough evidence yet that 3D TBP or AI is reliable on its own or beneficial as a support tool. Given the small samples and lack of blinded trials, further studies are needed to explore and improve the diagnostic capacities of 3D TBP and the possible integration of CNNs or other AI extensions and to examine the VECTRA 360WB compared to the usual standard of care.

## Introduction

Skin cancers (SCs) are a significant and growing global health concern. In the latest Global Cancer Statistics report, SC accounted for approximately 1.5 million of cancer cases and around 128,000 of the cancer fatalities worldwide [1]. In Belgium, SC accounted for nearly 44,000 of new cancer diagnoses, making it the most prevalent cancer. The most common types of SC are basal cell carcinoma (BCC) with an occurrence of 73.2%, followed by squamous cell carcinoma (SCC) with 18.8% and melanoma with 8% [2]. These percentages are comparable to other countries with a predominantly fair-skinned population [1,3].

An increase in the average annual percentage change was seen for all SC types in 2018, with rates rising by 9% for BCC, 7% for SCC, and 5% for melanoma. By 2030, a doubling of new diagnoses of BCC and SCC is expected. This increase is substantially higher than previous estimates based on population aging [2].

The prognosis for most SC is rather favorable. In Belgium, an increase in the 10-year relative survival rate is seen for all types of SC since 2004 [2]. The prognosis for melanoma, however, is highly dependent on the stage at diagnosis. While the 10-year survival rate for stage 1 melanoma is nearly 100%, this decreases to 70% for stage 2, 60% for stage 3, and a mere 20% for stage 4 [2,3]. The different melanoma stages respectively accounted for 79.6%, 12.9%, 5.6%, and 2.0% of the new diagnoses in Belgium in 2023 [4]. Melanomas diagnosed at more advanced stages require more aggressive treatment, along with intensive monitoring and follow-ups [3,5].

A Belgian cost-analysis estimated the economic burden of SC at approximately €106 million ($124 million) in 2014. The majority of these costs (65%) were attributed to melanoma, primarily due to the number of excisions, hospitalizations, and expensive advanced treatments. If the rising incidence trend continues, it is expected that the economic burden of SC will triple and lead to a cumulative cost rise estimated at €3 billion (US $3.5 billion) by 2034 [2,6].

Overall, it can be said that for all SC and especially for melanoma, early diagnosis improves quality of life, reduces morbidity and mortality, but also patient anxiety and health care costs [7]. To optimize early detection, the 2022 European Melanoma Guideline recommends the use of total body photography (TBP) in addition to sequential digital dermoscopy imaging (SDDI) in high-risk populations. Both interventions have been proven cost-effective in multiple analyses [3,8-10].

TBP has witnessed an evolution from 2D to 3D imaging since 2015. The first commercial 3D TBP system, the VECTRA WB360 3D whole-body imaging system (Canfield Scientific, Parsippany, NJ, USA), was launched in 2017 [11]. The device allows rapid and qualitative imaging through 92 cameras that simultaneously capture nearly the totality of the skin, including curved surfaces. This is followed by the construction of a 3D avatar of the individual [7,11]. Additionally, the VECTRA WB360 can be complemented by an SDDI device, and dermoscopic images can be tagged to the VECTRA body map. When combined, the skin surface and its existing lesions can be documented and compared at every visit [7,11-13]. Lastly, the VECTRA WB360 includes an automated lesion visualization software that can independently identify, count, and assess lesions on 3D images. This is based on several parameters, including the longest diameter, contrast, border and color variation, hue, and naevus confidence. At this time, this software is available as research-only tool. The dermoscopy explainable intelligence (DEXI) score is an artificial intelligence (AI)–generated risk score that is based on the SDDI images of the naevi and that can be used to assist in the diagnosis of suspicious lesions. However, the tools regarding naevi count and the DEXI risk assessment tool for dermatoscopic images can only be used in clinical trials and are not yet validated for clinical application [14-17]. The full technical details on the VECTRA WB360 can be found via Canfield Scientific [18].

With the increasing use of 3D TBP, specifically with the VECTRA WB360, it is timely to review the current literature reporting on the clinical utility, advantages, and limitations of this emerging technology for SC screening.

## Methods

First, a PICO (population, intervention, comparison, outcome) framework was established by the research team to explore keywords, search terms, and existing systematic reviews. No similar reviews were found up to March 2024.

An electronic literature search was conducted on PubMed from December 2023 to March 2024. The search terms included 3D imaging, VECTRA WB360, melanoma, nonmelanoma skin cancer (NMSC), their synonyms, and associated entry terms (using the National Institutes of Health). The synonyms were linked with the “OR” operator, while the different categories were linked with the “AND” operator. Since the first prototype of the 3D TBP was introduced in 2015, a 10-year filter was used. For the full search string, see Multimedia Appendix 1. An additional electronic search was conducted to obtain data on Belgian epidemiology and health care costs concerning SC screening and management.

Two authors (FB and ED) independently screened the search results (698 results). For records that were considered relevant according to title and abstract screening, full-text papers were obtained. Inclusion and exclusion criteria were applied by the same 2 authors. Publications that used a device other than the VECTRA WB360 were excluded, as were papers reporting on new technology without further research or without added cases. After thorough screening of the papers and removal of duplicates, 11 papers remained. All materials were imported to Zotero for citation management. The form and content of the scoping review comply with the PRISMA-ScR (Preferred Reporting Items for Systematic Reviews and Meta-Analyses Extension for Scoping Reviews) checklist (Checklist 1).

## Results

### Study Description

**Figure 1.**
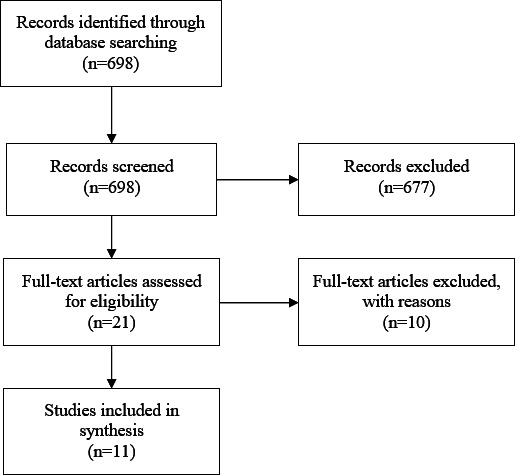
PRISMA (Preferred Reporting Items for Systematic Reviews and Meta-Analyses) flow diagram.

Given that 3D TBP is a recent innovation, only a limited number of studies are available (Figure 1). Five out of the 11 papers included in this review reported results from the Mind Your Moles (MyM) protocol by Koh et al[19]. This was the first study to evaluate 3D TBP in context of melanocytic naevi. The study was based on the general Australian population and consisted of nearly 200 participants from different risk groups. All participants were between the age of 20 and 69 years and had at least 1 naevus. All attended the clinic for an in-person skin examination, 3D TBP, and SDDI of lesions that were suspicious or >5 mm. This was repeated every 6 months over a period of 3 years with the purpose of monitoring changes in naevi [19]. These five papers included a report on the final outcomes [17], performance of the lesion visualizer [14,15,20], and study participant experiences [21]. An overview of the characteristics of the different studies can be found in Table 1.

**Table 1.** Characteristics of publications derived from the Mind Your Moles protocol.

Authors of publication	Soyer et al [17]	Jayasinghe et al [20]	Betz-Stablein et al [15]	Betz-Stablein et al [14]	Horsham et al [21]
Country	Australia	Australia	Australia	Australia	Australia
Year of publication	2023	2023	2022	2022	2022
Study design	Prospective population-based cohort study	Prospective population-based cohort study	Retrospective population-based cohort study	Retrospective population-based cohort study	Prospective population-based cohort study
Population type	General population	General population	General population	General population	General population
Data collection	December 2016 to February 2020	December 2016 to February 2020	December 2016 to February 2020	December 2016 to February 2020	December 2016 to February 2020
Participants, n	164	156	20 selected out of 163 total body images	92 total	149
Sex, %	Male: 58 Female: 42	Male: 63 Female: 37	Male: 61 Female: 59	Training Male: 62 Female: 48 Testing Male: 50 Female: 50	Male: 63.1 Female: 36.9
Age (years), median (range)	Not reported (18-70)	55 (23-70)	57 (25-72)	Training: 55 (23-69) Testing: 57 (37-67)	55 (23-70)
Training and testing images, n	Not applicable	Not applicable	Training: 14 Testing: 6	Training: 82 Testing: 10	Not applicable
Lesions, n	250	Not reported	Training: 882 cherry angioma 168,180 nonangiomas Testing: 334 cherry angioma 56,515 nonangiomas	62,610 Training: 57.742>2 mm 5106 naevi 52,636 nonnaevi Testing: 4.868>2 mm 520 naevi 4348 nonnaevi	Not applicable
Automated count	No	Yes	Yes	Yes	Not applicable
Lesion type	Suspicious lesions for MSC^a^ and NMSC^b^	Naevus	Cherry angioma	Naevus	Not applicable
Clinical examination	Yes by study clinician	Yes	20 participants	10 test participants	Yes
Use of 3D TBP^c^	3-year FU^d^ 6-month intervals	3-year FU 6-month intervals	One time No FU	One time No FU	3-year FU 6-month intervals
Fitzpatrick type	1‐4	1‐4	1‐4	1‐4	1‐4
SDDI of lesions	Yes when suspicious OR >5 mm	No	No	No	Not applicable
Histopathology	234 lesions	No	No	No	Not applicable
Lesion visualizer	No	Yes: >2 mm AND <100 naevi	Yes: >1 mm OR>2 mm	No	Not applicable
CNN^f^	No	No	No	Yes: >2 mm OR >5 mm	Not applicable
Conclusion	3D TBP results in diagnosis of a high number of NMSC and their precursors in the general population	3D TBP is an objective tool for automated naevus count	Angioma detection and counts are possible with 3D TBP	Objective naevus detection and counts are possible with 3D TBP CNN	Majority is content, ±50% see barrier

a MSC: melanoma skin cancer.

b NMSC: nonmelanoma skin cancer.

c TBP: total body photography.

d FU: follow-up.

e SDDI: sequential digital dermoscopy imaging.

f CNN: convolutional neural network.

The 6 remaining studies included participants at a higher risk of either having or acquiring SC instead of the general population. From these studies, there was 1 case series that included 2 high-risk patients and 1 participant from the MyM study [12]. Different from the MyM study and a case report by Rayner et al [11], all other studies employed one-time imaging using the VECTRA WB360 [16,22-24]. An overview of the characteristics of the studies not derived from the MyM protocol can be found in Table 2.

**Table 2.** Characteristics of publications not derived from the Mind Your Moles protocol.

Authors of publication	Hobelsberger et al [22]	Marchetti et al [24]	Grochulska et al [12]	Rayner et al [11]	Cerminara et al [16]	Jahn et al [23]
Country	Germany	United States	Australia	Australia	Switzerland	Switzerland
Year of publication	2023	2023	2021	2018	2023	2022
Study design	Prospective cohort study	Single-center retrospective observational study	Case series	Case report	Prospective single-center observational cohort study	Prospective single-center comparative observational cohort study
Population type	Patients with suspicious lesions of NMSC^a^	High-risk population	2 cases from high-risk population and 1 general population	High-risk population	High-risk population	High-risk population
Data collection	April 2021 to June 2022	July 2015 to October 2021	January 2017 to October 2020	May 2016 to February 2017	January 2021 to August 2021	January 2021 to June 2021
Participants, n	129	35	3	1	143	114
Sex, %	Male: 60 Female: 40	Male: 66 Female: 34	Male: 67 Female: 33	Female: 100	Male: 52 Female: 48	Male: 49 Female: 51
Age (years), median (range)	77 (42-91)	64 (26-89)	67 (48-71)	50 (not available)	56 (22-85)	59 (22-85)
Lesions, n	182	23,538; 49 MSC^b^ and 22,489 others	3	1	1690	1204
Automated count	No	Yes	No	No	Yes	Not reported
Lesion type	NMSC	MSC	Naevus	MSC	Naevus	Naevus
Clinical examination	Yes	No	Not reported	Not reported	Yes	Yes
Use of 3D TBP	One time No FU^c^	One time No FU	FU after 5‐12 months	FU after 3 and 9 months	One time No FU	One time No FU
Fitzpatrick type	1‐3	“White patients”	Not reported	Not reported	1‐4	1‐4
SDDI of lesions	No	No	Yes, of suspicious lesion	Yes, of suspicious lesions	Yes, of suspicious lesions OR >3 mm	Yes, of suspicious lesions OR >3 mm
Histopathology	158 lesions	43 melanomas	2 lesions	1 lesion	75 lesions	61 lesions
Lesion visualizer	No	Yes, lesions >2 mm	No	No	Not reported	Not reported
CNN^e^	No	No	No	No	Yes	Yes
Conclusion	Diagnostic accuracy for 3D TBP^f^ is slightly lower than for dermoscopy in NMSC diagnosis	3D TBP software for automated analysis has a high accuracy to differentiate MSC from other skin lesions	3D TBP is a valuable tool alongside dermoscopy to assist clinicians	3D TBP and SDDI can potentially increase the diagnostic accuracy in melanoma screening	Dermatologists are more accurate than dermatoscopic CNN included in 3D TBP and 2D TBP. The novel DEXI^g^ score on 3D-CNN device outperformed the 2D-CNN and achieved comparable sensitivity with dermatologists	Patients rate 3D TBP as trustworthy

a NMSC: nonmelanoma skin cancer.

b MSC: melanoma skin cancer.

c FU: follow-up.

d SDDI: sequential digital dermoscopy imaging.

e CNN: convolutional neural network.

f TBP: total body photography.

g DEXI: dermoscopy explainable intelligence.

Multiple studies used SDDI but often chose different criteria for inclusion of (pigmented) lesions. For example, Cerminara et al [16] and Jahn et al [23] included all lesions >3 mm, while Soyer et al [17] opted for lesions >5 mm. In each study, suspicious lesions were captured regardless of their size. Two studies evaluated the efficacy of the lesion visualizer [20,24]. Two more recent studies investigated whether the addition of AI, in the form of convolutional neural network (CNN), could improve the performance of the current software [14,15]. One study focused on the CNN on SDDI level, namely the DEXI score [16]. Furthermore, some studies explored participant experience when using the VECTRA WB360 [21,23]. Others were a case series and a clinical perspective with a case report [11,12], Lastly, but important to mention, all studies included participants with fairer skin types (Fitzpatrick I-IV).

### Utility of the VECTRA WB360

#### On-Screen Assessment by a Clinician

Two studies compared histopathological outcomes to diagnoses made on-screen on 3D TBP images by dermatologists. In the first study, a clinician based their clinical diagnosis on the 3D TBP images combined with SDDI of different types of lesions [17]. The second study did not use SDDI and solely included lesions that were suspicious for NMSC [22].

The first study, conducted from 2016 until 2020 in Australia, was based on the MyM protocol. A senior dermatologist used teledermatology for the assessment and diagnosis of lesions. This involved the use of 3D TBP imaging and SDDI of lesions that were suspicious or >5 mm. Fifty‐six percent of participants (108/193) received a referral for cryotherapy, topical treatment, further clinical examination, biopsy, or excision. Of those with a referral for biopsy or excision, 85% (86/101) visited their practitioner and underwent the recommended management. This accounted for a total of 138 lesions which were histopathologically examined. Thirty-seven of the 61 lesions that were clinically suspicious for skin malignancy were histopathologically confirmed. Eight of the 77 lesions that seemed clinically benign, turned out to be malignant after histopathological examination. In conclusion, on-screen assessment of 3D TBP images resulted in the diagnosis of mostly NMSC (BCC n=36 and SCC n=3) and their precursors (n=25) in the general population. Of the 15 lesions that were on-screen clinically suspicious for melanoma, only 6 were histopathologic confirmed. The reported number needed to excise (NNE) was 3.0/1.0 [17].

The second study was a German prospective cohort study conducted between 2021 and 2022. They included 129 patients who had a clinically suspicious lesion for NMSC and had not yet undergone a biopsy. All patients underwent a clinical examination followed by examination by dermoscopy and 3D TBP. The 3D TBP images were interpreted separately by another clinician who was blinded to the results in dermoscopy. Both clinicians, in clinic and on screen, gave a specific diagnosis for 182 suspicious lesions and their grade of certainty for said diagnosis. The diagnoses made in-person and on-screen were compared to the histopathological results of 158 lesions. They concluded that compared to clinical examination with dermoscopy, 3D TBP had lower sensitivity for BCC (73% vs 79%, *P*=.73), higher sensitivity for SCC (81% vs 74%, *P*=.73), and lower sensitivity for in situ SCC (0% vs 33%, *P*=.13). Specificity of 3D TBP was lower than that of dermoscopy for BCC (77% vs 82%, *P*=.58) and for SCC (75% vs 84%, *P*=.06), and higher specificity for in situ SCC (97% vs 94%; *P*=.34). However, the differences in sensitivity and specificity were not statistically significant. The diagnostic accuracy increased when the clinician was more certain of their diagnosis [22].

Lastly, on-screen naevus versus nonnaevus identification was compared to in-clinic identification by the same clinician in 10 test participants. This was examined in a broader study (cf. infra), which was also based on the MyM protocol. The overall agreement was 90% (4868 naevi), and the Cohen kappa was 0.45, indicating a moderate agreement between both methods [14].

#### Utility of the Lesion Visualizer and Added CNN

Only 1 American study conducted by Marchetti et al [24] investigated the use of imaging processing techniques on 3D TBP images. They found that the lesion characteristics provided in the lesion visualizer software could be used to detect melanoma and accurately distinguish between melanoma and other benign dermal lesions. This proof-of-concept study retrospectively investigated 35 patients who were diagnosed with at least 1 melanoma and had available 3D TBP imaging captured within 90 days prior to their histopathological diagnosis. This accounted for a total of 43 histopathologically confirmed melanomas, 29 in situ and 14 invasive.

The different prediction model variables and their individual ability to distinguish melanoma from nonmelanoma were examined based on their AUC. The variables can be categorized into size (area, diameter), color (asymmetry, variation, lesion vs non-lesion contrast), border (jaggedness, asymmetry), and anatomic site. The VECTRA DermaGraphix software detected 22,538 lesions, all >2mm. All lesions without histopathologic diagnosis of melanoma within 90 days after 3D imaging were classified as nonmelanoma (22,489 lesions). The other 49 lesions were labeled as melanoma lesions. In a patient level analysis, the prediction model-based probability for each lesion was ranked from lowest to highest. Of the melanoma lesions, 7 (14%) had the highest predicted score among all lesions for an individual patient, 7 (14%) were in the 99th percentile, 12 (25%) in the 98th percentile, 12 (25%) in the 90-97th percentiles and 11 (22%) in the 60-89th percentiles. Of the lesions that scored below the 90th percentile, 85% were incorrectly segmented. Five melanomas were recognized as 2 or more distinct lesions by the software, and 1 was not detected.

The 15 variable prediction model achieved an area under the curve of 0.94, indicating that it has a great prediction power. The segmentation of lesions was a notable limitation for the accuracy of the software. The research group further concluded that using a model-based threshold associated with 95% sensitivity for melanoma detection, the model could reduce the number of lesions requiring clinical examination by 75% [24].

#### Efficiency of Added CNN

The next 3 studies revealed how a CNN can improve the diagnostic accuracy of the existing system to aid clinicians in the assessment of skin lesions. A CNN is a form of AI used primarily for image recognition and processing because of its ability to recognize patterns. However, it is not possible to compare these studies or CNNs since they were engineered, trained, and tested following different protocols [14,15,20].

**Figure 2.**
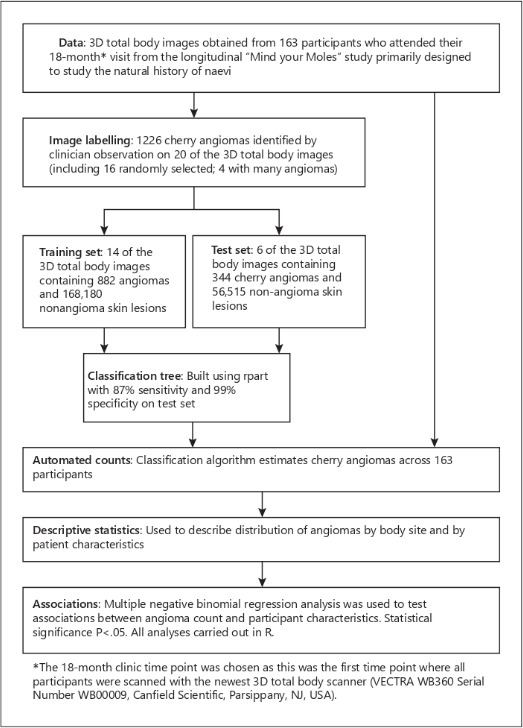
Study population and methodology. Adapted from Betz-Stablein et al [15].

Two of these studies were published by Betz-Stablein et al [14,15] and were derived from the MyM protocol in the general population. One study [15] trained a CNN on 14 3D TBP images and tested it on 6 3D TBP images. This CNN was used to detect, localize, and count cherry angiomas and thus distinguish them from other skin lesions. The study population and methodology can be found in Figure 2. A clinician identified all cherry angiomas on 20 participants, and these were then split 60:40 into a training and test set. The algorithm achieved a sensitivity of 87% and a specificity of 99% and was able to perform the requested task.

**Figure 3.**
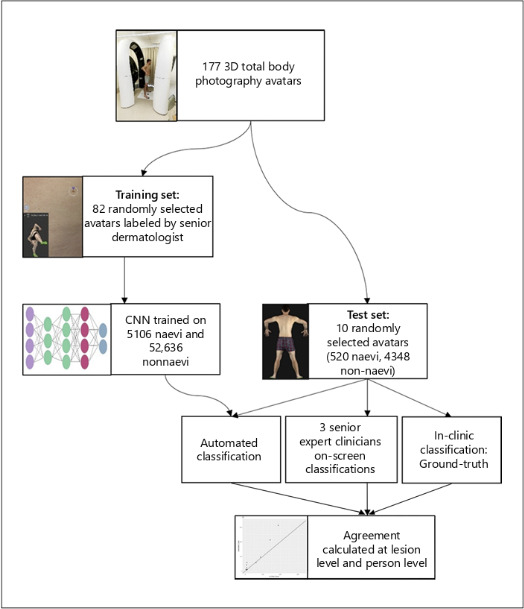
Methodology. Adapted from Betz-Stablein et al [14].

In the second study by Betz-Stablein et al [14], a CNN was trained on 82 participants (57,742 lesions >2 mm; 5106 naevi vs 52,636 nonnaevi lesions) and tested on 10 participants (4868 lesions >2 mm; 520 naevi vs 4348 nonnaevi lesions). An overview of the methodology can be found in Figure 3. This CNN was used to detect and count naevi (both >2 and >5 mm). The counts were compared to the in-clinic counts of a senior dermatologist (ground truth) and to on-screen counts of 3 expert clinicians on 3D TBP images. Nonnaevi lesions were mainly solar lentigines, seborrhoeic keratoses, or angiomas. The sensitivity of the 3D TBP CNN increased when only lesions >5 mm were included (79% for >2 vs 84% for >5 mm). The specificity stayed identical regardless of the diameter (91% for >2 and >5 mm). Comparing the CNN with the in-clinic assessment by the dermatologist resulted in a Cohen kappa of 0.56, indicating moderate agreement for naevi ≥2 mm and substantial agreement (0.72) for naevi ≥5 mm. The agreement was lower when participants had numerous seborrheic keratoses because of an overestimation of the number of naevi by the CNN. Using the findings of the previous study, a smaller study by Jayasinghe et al [20] utilized the VECTRA WB360 system to automatically count naevi >2 mm in 124 participants who had <100 naevi. Manual counts were used for the 32 participants with >100 naevi or >50 seborrhoeic keratosis. Their aim was to assess how naevi change during adulthood.

In a third study from a Swiss research group led by Cerminara et al [16], 2 different CNNs used on SDDI images were compared. Their objective was to examine the capabilities of AI in a real-world setting. In total, 1690 pigmented lesions in 143 participants at a high-risk of melanoma were used. All participants first underwent routine SC screening, including dermoscopy conducted by a dermatologist, who provided a dichotomous diagnosis (malignant vs benign) for each pigmented skin lesion. Patients then underwent TBP with both a 3D (VECTRA WB360) and 2D (2D-FotoFinder-ATBM) imaging system. After this, melanocytic lesions >3 mm and smaller suspicious lesions identified by dermatologists were captured with SDDI. The diagnostic accuracy of the CNN used on the SDDI images on the 3D TBP (DEXI score) and the CNN used on the SDDI images on the 2D TBP (FotoFinders Moleanalyzer Pro) was compared to clinical diagnoses of dermatologists and to histopathological examinations. It was found that when histopathological outcomes were used as a ground truth (75 lesions), dermatologists (without the aid of a CNN) achieved the highest specificity (92.3%). Second were dermatologists aided by a CNN (86.2%) and third a CNN without human interference (64.6% for 3D CNN and 40% for 2D CNN). However, the sensitivity of dermatologist, dermatologist+CNN, and the 3D CNN was identical (all 90%). It was concluded that 3D TBP CNN was superior to 2D TBP CNN (in both sensitivity and specificity).

This study also included a subanalysis in which the repetition rate of both CNNs was tested. The 3D TBP CNN had a higher repetition rate (0.89) than the 2D TBP CNN (0.79). In conclusion, the 3D CNN outperformed 2D CNN in the classification of melanocytic lesions and in the reproducibility of the scores. Although the 3D CNN demonstrated great scores, dermatologists continue to achieve higher specificity.

### Reported Advantages of 3D TBP

The main reported advantage of the VECTRA WB360 system is the comfortable, rapid, and noninvasive acquisition of high-resolution images that are used to create a 3D representation of the patient. This allows clinicians to assess and objectively compare the totality of the skin surface over time. This is especially important in melanoma screening, given that the evolution and appearance of new moles and lesions are important for the early detection of (de novo) SC [8,12-15,21]. By this, dermatologists know which naevi they should be aware of and should be carefully examined with dermatoscopy. The attached dermatoscope and the lesion visualization software allow the integration and assessment of dermoscopy images onto the 3D avatar. This enables a detailed and objective comparison of individual lesions. Although even without SDDI, the resolution of the 3D images is high enough to see important changes in lesions, especially when >5 mm. This is also shown in the publication of Grochulska et al where in one case a lesion showed asymmetric changes and in a second case a new lesion appeared [12].

When the consumer experience was explored in the 2 studies, it was concluded that 94% (140/149) of participants find 3D TBP a comfortable examination and 98% (145/148) would recommend it. The majority of participants have a high acceptability and confidence in the new technology, and they also report a reduction in melanoma-related anxiety [21,23].

Other reported potential benefits of the system include AI-assisted assessment of lesions in the future; introduction of automated, standardized, and timesaving naevus counts; increase in diagnostic confidence in clinicians; and further evolution of teledermatology with a potential decrease in waiting lists as a result [11,14].

### Reported Limitations of 3D TBP

Despite being presented as having the potential to be a great innovation, 3D TBP holds significant shortcomings. The most inconvenient one is the absence of visualization of the foot soles, scalp, and parts of the genital region in general [11,22]. In different trials, a small number of pigmented lesions were not detected or appeared as erythema on the 3D TBP images [22,24]. It is important to note that despite the current resolution of the images, the system is not able to replace a dermatological examination [11,22].

The algorithm performed poorly on participants with many seborrhoeic keratoses; however, this population can easily be identified in clinic and flagged for manual counts [14]. There is also a possibility that 3D TBP increased the number needed to excise due to a more prompt decision to perform a biopsy or excision when changes are seen, particularly in the younger population where changes are rather common and insignificant [11].

In the consumer experience study by Horsham et al [21], 6.7% (10/149) of the participants stated that they would not pay for 3D TBP examination and 2% (3/148) would not recommend it. They also found that 50% (74/149) of the study population remarked obstacles, mainly concerning the (digital) privacy, the high cost, and a lack of trust to detect and monitor small lesions.

Lastly, for clinicians, an important disadvantage is the practical and logistical implications of the VECTRA WB360. The system takes up a great amount of space and needs specialized information technology management and maintenance, leading to a high cost [11].

### Ongoing Studies

Three protocols centered around 3D TBP were found on the online databases. However, no results were found up to March 2024.

The first is a new prospective population-based cohort study protocol by the Australian research team of MyM [25]. These authors want to investigate whether 3D TBP can be used for melanoma imaging and diagnosis on 15,000 participants from the general population. An implementation of a CNN, a cost-analysis, and consumer experience are also included. The second protocol was released in 2019 and is by the same Australian research group under Primiero et al [13]. This is the only 2-arm randomized controlled trial protocol in a high-risk melanoma cohort, in which the authors compare standard clinical care to 3D TBP with SSDI. Another promising protocol for a randomized controlled trial in high-risk patients (so-called IMAGE-trial) was published by Yan et al [26]. This study will compare the proportion of false positives and false negatives with a standard clinical examination and 2D TBP and 3D TBP (with the VECTRA.WB360). Both arms will include the use of SDDI on suspicious lesions and up to 20 lesions of >4 mm. Furthermore, they will also assess economic impact, health-related quality of life, and diagnostic performance.

## Discussion

This review provides an overview on the current experience, outcomes, advantages, and limitations of 3D TBP with the VECTRA WB360 in a mainly fair-skinned population (Australia and Europe). Currently, SC is the most diagnosed form of cancer in Belgium, and the incidence of both melanoma and NMSC is increasing, as well as the cost of SC management. Given these trends, it is crucial to explore innovative methods for early SC detection and more efficient, time-saving follow-up strategies. Also, with the increasing use of 3D TBP, it is timely to explore the reported outcomes to date.

While the heterogeneity of study designs restricted a quantitative analysis of results, a scoping synthesis found agreeance over general outcomes and conclusions. The VECTRA WB360 shows significant promise for mapping and monitoring the evolution of the skin surface and individual lesions through additional SDDI. This system not only allows new lesions to be detected but also enables objective comparison of pre-existing lesions (on a macroscopic and a dermoscopic level). Research shows that despite the lower resolution of 3D TBP than dermatoscopy, it is suitable to detect changes in lesions; however, dermoscopy is still generally required to diagnose a suspected melanoma. All these tools are enabling and facilitating teledermatology.

The lesion visualizer was examined in only 3 studies, where it was used to differentiate melanoma from benign lesions and count naevi and cherry angiomas [14,15,24]. The most significant limitation so far is its inaccurate segmentation of melanoma. In the study by Marchetti et al [24], 11% of melanomas (5 out of 44) were incorrectly segmented, raising concerns that some melanomas could be missed. Additionally, the study suggests that using a model-based threshold associated with 95% sensitivity for melanoma detection could reduce the number of lesions requiring clinical examination by 75%. However, this remains a hypothesis that has yet to be validated. If confirmed, this could be particularly useful in clinical practice. In the study by Betz-Stablein et al [14], the Cohen kappa of the CNN-based count to the gold standard clinical count was 0.56, indicating that while the lesion visualizer can provide an estimate of naevi numbers, it is not reliable for tracking the development of new naevi. Furthermore, it should be noted that in another study by Betz-Stablein et al [15], only clinicians identified all cherry angiomas on 20 participants, which does not provide sufficient evidential strength. While counting cherry angiomas could demonstrate the lesion visualizer’s ability to differentiate between cherry angiomas and naevi, which is important for the naevus count, its clinical utility remains limited. In the future, addition of CNN may improve efficiency by identifying new and changing lesions for closer inspection, and this can prove to be particularly useful for people with many naevi. It may also be used as a tool for imaging technicians to flag which lesions are important to take additional dermoscopy images of for further review by a dermatologist.

Only one study looked at the DEXI score which is available as a research tool only on SDDI images. When using the histology as ground truth, the dermatologist, dermatologist+AI, and the DEXI score all had the same sensitivity of 90%. The specificity of the dermatologist is the highest at 92.31%, then dermatologist+AI at 86.15%, and finally DEXI score with 64.62% [16]. All methods have the same chance of missing lesions, but the difference lies in labeling benign lesions as malignant. This shows that the use of DEXI score alone would lead to a lot more excisions which were not necessary in the first place. It should be noted that more patients were examined by beginner dermatologists than seniors. In a clinical setting, it is also not feasible to take dermatoscopic images of all the naevi of a patient.

Although initial results show potential, the VECTRA system cannot yet be used without clinician supervision. At present, the software and AI tools cannot replace a trained clinician’s skin examination. In the future, AI could be used as a support tool during a skin exam to help the dermatologists with their clinical decision-making process. Especially the lack of specificity, compared to dermatologists seen in multiple studies, needs to be addressed. It is likely that imaging resolution and technology will improve with time, and new CNNs such as lesion classifiers are likely to be next. These developments can strengthen specificity and accuracy, and the system will potentially become a more efficient and reliable tool for SC screening and other tasks. But before this can become a widely implemented tool, its safety, cost effectiveness, and added value to the standard of care must be proven. The AI tool is so new that long-term health outcomes cannot yet be discussed.

It should be noted that 5 out of the 11 studies were based on the same study protocol and participants [14,15,17,20,21]. This makes the available research population less diverse. Moreover, the inclusion of only 200 participants for a population-based study is insufficient, especially in a large country with a high prevalence of SC like Australia. Also, no control group was available, which makes the results of these studies less robust. A control group is essential when evaluating new technologies. This allows for a comparison to the usual standard of care and to evaluate any added benefits.

The study by Soyer et al [17] concludes that 3D total body imaging results in the diagnosis of a high number of keratinocyte cancers. However, this finding is unsurprising, as keratinocyte SC are the most common type and are diagnosed more frequently than melanoma. Additionally, the lack of a control group makes it unclear whether this approach resulted in a higher detection rate compared to standard care. Another limitation is that the study does not specify who the study clinicians are. The decision on which lesions are clinically suspicious and therefore referred for tele review by an experienced dermatologist was left to these clinicians. If they were less experienced or lacked training in dermosocopy, it is possible that certain lesions were missed. Furthermore, the study reports that 8 clinically benign lesions turned out to be malignant. Of these, 7 were first diagnosed as actinic keratosis and 1 as a naevus. It remains unclear why excision or biopsy was recommended for these lesions. Was there already diagnostic uncertainty, or were other factors influencing these decisions?

The publications that were not part of the MyM protocol also included few patients [11,12,16,22-24]. One showed a clinical example and was speculative about the future, and another was a more illustrative case series. This does not bring an added value to the proof of safety and efficiency of the new technology [11,12]. The study by Hobelsberger et al [22] also had several limitations. Firstly, there was only 1 clinician who performed the interpretation of the 3D images, and another who conducted the clinical examination. When the interpretations of only 2 individuals are compared, does the study say a lot about the differences between the methods or does it say more about the diagnostic skills of the clinicians? Bigger studies with more clinicians are needed to give more representative results. Secondly, the authors mention that it is important to minimize the number of missed lesions, for this a high sensitivity is needed. Currently, the sensitivity and specificity of diagnosis of NMSC on a 3D image are lower than that of clinical examination aided by dermatoscopy, although not statistically significant. This may result in a greater number of missed lesions. Of course, we should also consider that this study is used in a setting of teledermatology. It is often quicker and more straightforward to conduct an in-person examination instead of looking at the whole skin on a 3D image, but this is not always possible.

Because the available research is limited and heterogeneous, it will be important to perform further studies. A new study with a bigger cohort that tests the DEXI-score of naevi should also be conducted. At present, only 1 study has examined this, which is not enough to prove the accuracy of AI. The distribution between beginner and senior dermatologists in studies should be even. To further test the capabilities of CNN, a study in which sequential 3D TBP at 1, 2, and 3 years is performed to detect new and altered naevi by the CNN can be conducted. New and possibly changed naevi should also be checked by a dermatologist. A margin of error can be determined. New studies should also include randomized controlled trails to further examine the VECTRA WB360 potential in a real-world clinical setting. Numerous promising potential benefits are mentioned by the researchers such as increased diagnostic confidence and decrease of benign/malignant ratio, but at the moment, these all are not validated. In addition, the impact on survival rates and morbidity needs to be examined to prove the real-world impact of the system. It will be also important to compare examinations aided by the VECTRA WB360 to the normal standard of care to see if it brings an added value.

Possible obstacles for the wider implementation of 3D TBP could relate to the high cost of the system and thus the imaging sessions (for both clinicians and patients), medico-legal arrangements, difficulties in terms of privacy legislation (General Data Protection Regulation for Europe), unsuitability for people with certain disabilities/medical conditions, and the consumers’ view on new technology.

In conclusion, while it is important to embrace new technologies, their adoption must be approached with appropriate caution and critical evaluation. Though the system may allow faster detection of malignancies, relying solely on AI risk assessment tools may lead to lesions being incorrectly assumed as suspicious due to change detection, particularly in the younger population where naevus changes are frequent and typically benign. This could result in unnecessary biopsies and excisions and consequently in increasing costs and patient anxiety and discomfort. Therefore, it is important to make decisions about treatment and excision in the context of the whole patient, and not just the VECTRA images. Further clinical trials, including all Fitzpatrick skin types and a wide range of lesion sizes, are necessary to prove the clinical relevance and the autonomy of the VECTRA WB360.

### Conclusion

In this review, the current knowledge, outcomes, advantages, and disadvantages of 3D TBP with the VECTRA WB360 and the integrated lesion visualization software are presented. While the VECTRA 3D TBP holds substantial promise for the early detection and monitoring of SC, its application cannot yet replace the expertise of trained clinicians. Although the lesion visualizer and DEXI score offer potential enhancements, they also pose risks, including a significant increase in unnecessary excisions due to lower specificity. Despite these promising results, expert overview is still recommended and superior, since there is not enough evidence yet that 3D TPB or AI is reliable on its own or beneficial as a support tool. Given the small samples and lack of blinded trails, further studies are needed to explore and improve the diagnostic capacities of 3D TBP and the possible integration of CNNs or other AI extensions. It will also be important to examine the VECTRA 360WB compared to the usual standard of care.

## Supplementary material

10.2196/68510 Multimedia Appendix 1 Search terms.

10.2196/68510 Checklist 1 PRISMA-ScR checklist.

## Abbreviations

AI: artificial intelligence
BCC: basal cell carcinoma
CNN: convoluted neural network
DEXI: dermoscopy explainable intelligence
MyM: Mind Your Moles
NMSC: nonmelanoma skin cancer
SC: skin cancer
SCC: squamous cell carcinoma
SSDI: sequential digital dermoscopy imaging
TBP: total body photography

## References

[1] Bray F, Laversanne M, Sung H (2024). Global cancer statistics 2022: GLOBOCAN estimates of incidence and mortality worldwide for 36 cancers in 185 countries. CA Cancer J Clin.

[2] Boonen B, Goossens M, Schutter H, Walle L (2021). Huidkanker in België [Report in Dutch]. https://www.lymfklierkanker.be/Uploads/Huidkanker%202021.pdf.

[3] Garbe C, Amaral T, Peris K (2022). European consensus-based interdisciplinary guideline for melanoma. Part 1: diagnostics: update 2022. Eur J Cancer.

[4] (2023). Melanoma cancer fact sheet. https://kankerregister.org/sites/default/files/2025/2025_BE_CFS2023_APP_MELANOMA_2.pdf.

[5] Gershenwald JE, Scolyer RA, Hess KR (2017). Melanoma staging: evidence‐based changes in the American Joint Committee on Cancer eighth edition cancer staging manual. CA Cancer J Clin.

[6] Pil L, Hoorens I, Vossaert K (2016). Burden of skin cancer in Belgium and cost-effectiveness of primary prevention by reducing ultraviolet exposure. Prev Med.

[7] Mahumud RA, Janda M, Soyer HP, Fernández-Peñas P, Mar VJ, Morton RL (2022). Assessing the value of precision medicine health technologies to detect and manage melanoma. Med J Aust.

[8] Salerni G, Carrera C, Lovatto L (2012). Benefits of total body photography and digital dermatoscopy (“two-step method of digital follow-up”) in the early diagnosis of melanoma in patients at high risk for melanoma. J Am Acad Dermatol.

[9] Truong A, Strazzulla L, March J (2016). Reduction in nevus biopsies in patients monitored by total body photography. J Am Acad Dermatol.

[10] Watts CG, Cust AE, Menzies SW, Mann GJ, Morton RL (2017). Cost-effectiveness of skin surveillance through a specialized clinic for patients at high risk of melanoma. J Clin Oncol.

[11] Rayner JE, Laino AM, Nufer KL (2018). Clinical perspective of 3D total body photography for early detection and screening of melanoma. Front Med (Lausanne).

[12] Grochulska K, Betz-Stablein B, Rutjes C (2022). The additive value of 3D total body imaging for sequential monitoring of skin lesions: a case series. Dermatology (Basel).

[13] Primiero CA, McInerney-Leo AM, Betz-Stablein B (2019). Evaluation of the efficacy of 3D total-body photography with sequential digital dermoscopy in a high-risk melanoma cohort: protocol for a randomised controlled trial. BMJ Open.

[14] Betz-Stablein B, D’Alessandro B, Koh U (2022). Reproducible naevus counts using 3D total body photography and convolutional neural networks. Dermatology (Basel).

[15] Betz-Stablein B, Koh U, Edwards HA, McInerney-Leo A, Janda M, Soyer HP (2022). Anatomic distribution of cherry angiomas in the general population. Dermatology (Basel).

[16] Cerminara SE, Cheng P, Kostner L (2023). Diagnostic performance of augmented intelligence with 2D and 3D total body photography and convolutional neural networks in a high-risk population for melanoma under real-world conditions: a new era of skin cancer screening?. Eur J Cancer.

[17] Soyer HP, O’Hara M, V Silva C (2023). Skin cancer excisions and histopathology outcomes when following a contemporary population-based cohort longitudinally with 3D total-body photography. Skin Health Dis.

[18] VECTRA WB360 imaging system. Canfield Scientific.

[19] Koh U, Janda M, Aitken JF (2018). “Mind your Moles” study: protocol of a prospective cohort study of melanocytic naevi. BMJ Open.

[20] Jayasinghe D, Koh U, Plasmeijer EI (2023). The dynamic nature of naevi in adulthood: prospective population-based study using three-dimensional total-body photography. Br J Dermatol.

[21] Horsham C, O’Hara M, Sanjida S (2022). The experience of 3D total-body photography to monitor nevi: results from an Australian general population-based cohort study. JMIR Dermatol.

[22] Hobelsberger S, Steininger J, Laske J (2024). Clinician’s ability to identify non-melanoma skin cancer on 3D-total body photography sectors that were initially identified during in-person skin examination with dermoscopy. Dermatology (Basel).

[23] Jahn AS, Navarini AA, Cerminara SE (2022). Over-detection of melanoma-suspect lesions by a CE-certified smartphone app: performance in comparison to dermatologists, 2D and 3D convolutional neural networks in a prospective data set of 1204 pigmented skin lesions involving patients’ perception. Cancers (Basel).

[24] Marchetti MA, Nazir ZH, Nanda JK (2023). 3D Whole-body skin imaging for automated melanoma detection. J Eur Acad Dermatol Venereol.

[25] Koh U, Cust AE, Fernández-Peñas P (2023). ACEMID cohort study: protocol of a prospective cohort study using 3D total body photography for melanoma imaging and diagnosis. BMJ Open.

[26] Yan MK, Cust AE, Soyer HP (2023). Study protocol for a randomised controlled trial to evaluate the use of melanoma surveillance photography to the Improve early detection of MelanomA in ultra-hiGh and high-risk patiEnts (the IMAGE trial). Trials.

