# Supplementary material for: 3D Total Body Photography as a Promising Innovation for Early Skin Cancer Detection: Scoping Review
Source: JMIR Dermatol. 2025 Dec 17;8:e68510. doi: 10.2196/68510 (PMC12710984; doi:10.2196/68510)
Supplement: Multimedia Appendix 1 [file derma-v8-e68510-s001.docx]

## **Appendix 1**

(“3D total body photography” OR “Vectra WB360” OR “3D whole body” OR “Three-dimensional” OR “Three dimensional” OR “Imaging, Three-dimensional” OR ("Imaging, Three-Dimensional"[MESH])) AND (“Skin neoplasms” OR “melanoma” OR ("Melanoma/diagnosis"[MESH]) OR ("Melanoma"[Mesh]) OR ("Skin Neoplasms"[Mesh]))
